# Gestational Diabetes Mellitus: Long-Term Consequences for the Mother and Child Grand Challenge: How to Move on Towards Secondary Prevention?

**DOI:** 10.3389/fcdhc.2020.546256

**Published:** 2020-11-04

**Authors:** Eyal Sheiner

**Affiliations:** Department of Obstetrics and Gynecology, Soroka University Medical Center (SUMC), Ben-Gurion University of the Negev (BGU), Beer-Sheva, Israel

**Keywords:** gestational diabetes mellitus (GDM), diabetes, long-term cardiovascular disease, pregnancy, offspring, obesity, long-term morbidity

## Introduction

Gestational diabetes mellitus (GDM) is defined as glucose intolerance that begins during pregnancy (1). GDM occurs when the body is no longer able to adapt to its new circumstances, and the endocrine system is unable to produce sufficient insulin (2). GDM affects approximately 4-10% of pregnancies in the United States annually, depending on the characteristics of the population studied, and the diagnostics utilized (3, 4). It is clear that GDM is associated with adverse immediate pregnancy outcomes. These include an increased risk for gestational hypertension, preeclampsia and cesarean section (5). Additionally, fetal complications of GDM pregnancies include increased risk of macrosomia, shoulder dystocia, neonatal hypoglycemia and hyperbilirubinemia, and operative delivery (6).

However, recent research has shown that GDM continues to affect maternal and neonatal health long after the index pregnancy.

## Long-Term Consequences to the Mother

### Type 2 Diabetes Mellitus (T2DM)

Women with previous GDM have been shown to exhibit substantially increased risk for the development of T2DM, (7–10). A systematic review of 20 collated research studies found that GDM patients have an approximately 7-fold increase in risk of T2DM compared to no-GDM pregnancies (7). A population-based study found that 18.9% of women with previous GDM developed T2DM within 9 years after the index pregnancy; the rate was only 2% in the comparison group of women without GDM (2.0%). The rate of T2DM development increased in the first nine months postpartum, and 3.7% of patients had developed T2DM within nine months (8).

### Cardiovascular Disease

GDM increase postpartum risk of metabolic syndrome and cardiovascular disease. Metabolic syndrome is characterized by several risk factors, including central obesity, hypertension, insulin resistance, and dyslipidemia (11). Several studies have demonstrated an association between GDM and risk of subsequent maternal cardiovascular morbidity (12–14). Women with previous GDM have also an increased risk of cardiovascular risk factors such as hypertension, dyslipidemia, obesity, and metabolic syndrome (14–16). Specifically, GDM was found to be an independent risk factor for long-term maternal risk of noninvasive diagnostic procedures, simple cardiovascular events, and cardiovascular hospitalizations (12). Bo et al. followed women with GDM 6.5 years postpartum, and found they had significantly higher levels of vascular endothelial dysfunction markers than women with normal pregnancies. The authors concluded that GDM mothers experience a higher risk of future cardiovascular diseases than do normoglycemic mothers (17). Moreover, another study reported that when slight glucose intolerance (regardless of overt GDM) exists, a dose-response effect can be seen between glucose level during pregnancy and postpartum atherosclerotic morbidity (18).

### Malignancies

Women with a history GDM might have a higher future long-term risk for the development of malignancies: hospitalizations due to malignancies years after postpartum were increased in women with GDM. A significant association was documented between GDM and risk of developing ovarian, endometrial, and/or breast cancer (19). Other studies showed a positive association between GDM and higher glucose levels during pregnancy and the risk of breast cancer (20, 21). Perrin *et al.*, with a follow-up of 28–40 years, documented five cases of pancreatic cancer in women with GDM history, and an adjusted relative risk of 7.1 was documented (21). Similarly, another study indicated that GDM women were more likely to be diagnosed with pancreatic cancer (22).

### Ophthalmic Disease

GDM has also been reported to be a significant risk factor for long-term ophthalmic morbidity: Women with GDM history had significantly higher incidence of ophthalmic morbidity (e.g., glaucoma, diabetic retinopathy, retinal detachment) compared to controls (23). A significant number of women who experience GDM will develop T2DM years following postpartum, and as such retinopathy will effect up to 20% of them (24).

### Renal Disease

GDM seems like a significant risk factor for long-term renal morbidity. The most common future renal diagnoses were hypertensive renal disease without renal failure, hypertensive renal disease with renal failure, chronic renal failure, and end-stage renal disease (25).

## Long-Term Consequences to the Offspring

### Glucose Intolerance

Maternal diabetic intrauterine environment is strongly associated with T2DM development in the offspring. In a multiethnic population 30.4% of youth with T2DM had been exposed to maternal diabetes, compared to 6.3% of nondiabetic youth controls (26). In another cohort of obese adolescents 31.1% of obese children with normal glucose tolerance who had been exposed to GDM developed impaired glucose tolerance/diabetes over a relatively short follow-up period (avg. < 3 years). The results indicate that offspring of mothers with GDM history have at least 5 times greater risk of developing impaired glucose tolerance than those not exposed to gestational diabetes (27). Similarly, 21% of youth with either T2DM or pre-diabetes (impaired glucose tolerance or impaired fasting glucose) were offspring of women with diet-treated GDM, compared to 4% of women from the background population (28). The Pima have exceptionally high rates of obesity and T2DM. T2DM prevalence in Pima children is up to 6 times greater in those with diabetic or prediabetic mothers; T2DM in childhood and adolescence occurs almost exclusively among the offspring of diabetic and prediabetic mothers (29). There is evidence that the higher frequency of diabetes and obesity in the offspring of diabetic Pima women is not only due to genetic susceptibility to obesity and diabetes. Studies including sibling pairs with one sibling born before the onset of maternal diabetes, and one after, have brought interesting data: Being born after the mother developed diabetes led to a significantly higher risk of diabetes in offspring (30, 31).

### Obesity

The relationship between childhood elevated BMI and maternal diabetes was examined in a comprehensive metaanalysis, and a strong relationship was found between prenatal exposure to maternal diabetes and increased childhood BMI (32). Abokaf et al. found that the rate of obesity following in-utero exposure to GDM was as high as 4.9% following diet controlled GDM and 7.8% following GDM uncontrolled by diet. The rates of obesity in offspring of non-GDM women was as low as 1.8% (P<0.001) (33). The association between GDM and obesity of the offspring was established in other cohorts (34–36) and reviews (37).

### Endocrine Morbidity

A recent population-based study found a significant association between exposure to GDM and the risk for long-term endocrine morbidity in the offspring (33). During the study period 231,271 deliveries met the inclusion criteria, of which 12,642 deliveries (5.4%) were diagnosed with GDM. The incidence rate of diabetes mellitus, overweight and obesity among children was significantly higher in the GDM group. The rates of hypothyroidism were comparable between the groups. Using a GEE model, controlling for confounders such as maternal age, follow up-time, obesity and birthweight, in-utero exposure to diet controlled GDM (adjusted OR = 2.1; 95% CI 1.7–2.7; P < 0.001) and especially to GDM uncontrolled by diet (adjusted OR = 3.1; 95% CI 2.2–4.4, P < 0.001) were found as risk factors for long-term endocrine disease during childhood including diabetes overweight and obesity (33).

### Cardiovascular Morbidity

A significant association was recently noted between GDM and the rate of cardiovascular hospitalizations of the offspring (38). Nevertheless, the risk was extremely low: 0.97% for GDM A2 vs. 0.57% for GDM A1 vs. 0.33% for no GDM, respectively; p < 0.001.

### Neurodevelopmental Outcome and Neuropsychiatric Morbidity

Previous literature suggests convincing evidence that offspring of diabetic mothers are at risk of impaired neurodevelopmental outcome (39). A population-based study conducted in southern Israel investigated long-term neuropsychiatric morbidities in offspring exposed to GDM. Neuropsychiatric illnesses included in this study were autistic spectrum disorder, eating disorders, cerebral palsy, obstructive sleep apnea, epilepsy, and infantile spasms (40). During the study period 231,271 deliveries met the inclusion criteria; 5.4% of the births were to mothers diagnosed with GDM (n = 12,642), of these 4.3% had GDM type A1 (n = 10,076) and 1.1% had GDM type A2 (n = 2566). During the follow-up period, a significant linear association was noted between the severity of the gestational diabetes (no gestational diabetes, gestational diabetes mellitus A1, gestational diabetes mellitus A2) and neuropsychiatric disease of the offspring (1.02% vs 1.36% vs 1.68%, respectively, P <.001). Additionally, children exposed to GDM who developed neuropsychiatric disease did so at a younger age than their unexposed counterparts: A Kaplan-Meier curve demonstrated that children born to women with GDM had higher cumulative incidence of neuropsychiatric morbidity. Using a generalized estimating equation multivariable logistic regression model, controlling for time-to-event, maternal age, gestational age at delivery, maternal obesity, maternal preeclampsia and fertility treatments, maternal GDM was found to be an independent risk factor for long-term neuropsychiatric disease of the offspring.

### Ophthalmic Disease

A recent study investigated whether children born to mothers with GDM are at increased risk to develop pediatric ophthalmic morbidity. Offspring of patients with GDM treated by medication had a higher cumulative incidence of ophthalmic morbidity when compared to the other groups (Kaplan-Meier log rank test p = 0.038). GDM treated by medication was found to be an independent risk factor for long-term ophthalmic morbidity, in a cox multivariable model (adjusted HR: 1.5, 95% CI: 1.05-2.1, p = 0.025). Authors concluded that GDM treated by medication was associated with an increased risk for long-term pediatric ophthalmic morbidity (41).

## Grand Challenges

Worldwide, the prevalence of GDM in women of childbearing age has been on the rise (42).

As was reviewed in this article, solid data exists regarding the association between gestational diabetes mellitus (GDM) and long-term maternal cardiovascular disease. The rate of cardiovascular disease following GDM is about 9%. Likewise, several studies found an association between GDM and other maternal long-term complications such as renal, ophthalmic and even oncological diseases. Recent studies found an association between GDM and long-term complications to the offspring. While the risk for most morbidities is relatively low, although significant, the risk for endocrine morbidity of the offspring can reach 8%.

The grand challenges are how to use this data to move towards secondary prevention. Secondary prevention involves population at risk, i.e. women with GDM and their offspring that has not yet developed clinical signs and symptoms of the disease. Prevention programs should focus on secondary prevention of cardiovascular disease in patients following GDM, which deserve post-partum follow-up visits. This knowledge had recently led the International Federation of Gynecology and Obstetrics (FIGO) to recommend follow-up of all women with GDM 6–12 weeks after birth, and periodically thereafter, with screening for overt diabetes and cardiovascular risk factors (42). In an attempt to achieve consensus on uniform diagnostic criteria from this diversity and in response to the HAPO data (43) the International Association of Diabetes in Pregnancy Study Groups (IADPSG) convened a consensus panel who developed outcome-based criteria for the diagnosis of GDM (44). The panel considered a variety of possible diagnostic strategies, which were adopted by a variety of professional health-care bodies. Nevertheless, the FIGO guidelines (42) were recommended regardless of the definitions used for GDM.

In addition, secondary prevention should focus on offspring of GDM mothers for the prevention of obesity and diabetes of the offspring.

## Author Contributions

The author confirms being the sole contributor of this work and has approved it for publication.

## Conflict of Interest

The author declares that the research was conducted in the absence of any commercial or financial relationships that could be construed as a potential conflict of interest.
